# PROTOCOL: Exploring education to support vaccine confidence amongst healthcare and long‐term care staff amidst the COVID‐19 pandemic: A protocol for a living scoping review

**DOI:** 10.1002/cl2.1293

**Published:** 2022-12-07

**Authors:** Anna C. Reed, Maya Murmann, Amy Ramzy, Mary Scott, Becky Skidmore, Vivian Welch, Amy T. Hsu

**Affiliations:** ^1^ Bruyère Research Institute Bruyère Continuing Care Ottawa Canada; ^2^ Institute of Health Policy, Management and Evaluation, Dalla Lana School of Public Health University of Toronto Toronto Canada; ^3^ Independent Information Specialist Ottawa Ontario Canada; ^4^ School of Epidemiology and Public Health, Faculty of Medicine University of Ottawa Ottawa Canada; ^5^ Department of Family Medicine, Faculty of Medicine University of Ottawa Ottawa Canada; ^6^ Department of Clinical Epidemiology, Ottawa Hospital Research Institute The Ottawa Hospital Ottawa Canada

## Abstract

Despite the demonstrated effectiveness of vaccines, varying levels of hesitancy were observed among healthcare and long‐term care workers, who were prioritized in the roll out of COVID‐19 vaccines due to their high risk of exposure to SARS‐CoV‐2 transmission. However, the evidence around the measurable impact of various educational interventions to improve vaccine confidence is limited. The proposed scoping review is intended to explore any emerging research and experiences of delivering educational interventions to improve COVID‐19 vaccine confidence among health and long‐term care workforces. We aim to identify characteristics of both informal and formal educational interventions delivered during the pandemic to support COVID‐19 vaccine hesitancy. Using the guidance outlined by the Joanna Briggs Institute, we intend to search five databases including, Ovid MEDLINE and Web of Science, as well as grey literature. We will consider all study designs and reports in an effort to include a breadth of sources to ensure our review will capture preliminary evidence, as well as more exploratory experiences of COVID‐19 vaccine education delivery. Articles will be screened by three reviewers independently and the data will be charted, and results described narratively.

## 1 INTRODUCTION

The vaccination of healthcare workers, especially those who work in long‐term care settings, against COVID‐19 has been a top priority, given their high risk of COVID‐19 exposure and close contact with vulnerable populations (Government of Canada, 2020). Despite the demonstrated effectiveness of currently available vaccines, varying levels of vaccine hesitancy among both healthcare and long‐term care workers were observed during the rollout of COVID‐19 vaccines (Biswas et al., 2021; Desveaux et al., 2021; Harrison et al., 2021; Heyerdahl et al., 2022; McGarry et al., 2021; Sinha et al., 2021). Vaccine hesitancy, by definition, is influenced by several issues related to confidence, complacency, and convenience (Karafillakis et al., 2016). Vaccine hesitancy among healthcare workers is not new. In fact, drivers of vaccine hesitancy among healthcare workforces have been comprehensively studied and well‐described (Karafillakis et al., 2016). Vaccine safety, including risk of side effects, perceived lack of testing, and the safety and efficacy of newer vaccines are some of the most important concerns among healthcare workers, as well as mistrust in health authorities (Karafillakis et al., 2016).

Vaccine hesitancy is an intricate and context‐specific issue (SAGE Working Group on Vaccine Hesitancy, 2014) often requiring a multi‐dimensional approach to address it (Jarrett et al., 2015). According to a recent systematic review, efforts to respond to general vaccine hesitancy have been limited and disparate, varying significantly in the strategies introduced and corresponding effect size (Jarrett et al., 2015). Despite our understanding of why people, including healthcare and long‐term care workers, might be vaccine hesitant, the evidence around the measurable impact of various education strategies to improve vaccine confidence is limited, as few strategies have been rigorously or formally evaluated (Jarrett et al., 2015). Furthermore, given the novelty and unique circumstances surrounding the introduction of the COVID‐19 vaccines (i.e., in the context of a global pandemic and the need for rapid development, approval, and dissemination), prior strategies informed by research on adherence to the seasonal influenza vaccine (Dini et al., 2018; European Centre for Disease Prevention and Control, 2015; Hussain et al., 2018; Manuel et al., 2002; Oguz, 2019; Pereira et al., 2018; Schmid et al., 2017) may not be transferrable in the COVID‐19 context. Therefore, it could be important that educational strategies developed during the pandemic are specifically tailored to address hesitancy surrounding the COVID‐19 vaccines more specifically.

This scoping review is part of a broader research project seeking to minimize the gap between existing research and practice regarding the approach to developing and delivering educational interventions to improve COVID‐19 vaccine confidence, particularly among the healthcare and long‐term care workforces. As part of this research, we are also conducting an environmental scan in Ontario, Canada, that examines the delivery characteristics of COVID‐19 vaccine education offered to staff in long‐term care homes during the pandemic. This protocol outlines our review phase, which aims to explore and characterize both informal and formal educational interventions developed and delivered during the COVID‐19 pandemic to support COVID‐19 vaccine confidence. Furthermore, this review intends to identify how research is conducted on this topic, as well as any gaps in the available literature. This scoping review will be conducted following the guidance outlined by the Joanna Briggs Institute (Peters et al., 2020) and reported using the Preferred Reporting Items for Systematic Reviews and Meta‐Analysis Extension for Scoping Reviews (PRISMA‐ScR) (Tricco et al., 2018).

## 2 RESEARCH QUESTIONS

1. What educational interventions have been developed to encourage COVID‐19 vaccine uptake and support vaccine confidence during the COVID‐19 pandemic?
2. What are the characteristics of these interventions?
3. What characteristics, if any, could be applied to educational interventions delivered to health and long‐term care workforces?

## 3 INCLUSION CRITERIA

While our broader research is focused on exploring and understanding how educational strategies can be applied in health and long‐term care settings to support COVID‐19 vaccine confidence among these workforces, the scope of this review captures COVID‐19 vaccine educational interventions that were delivered to all populations in a variety of contexts. This was done for two reasons: (1) our trial searches that focused specifically on COVID‐19 vaccine educational interventions targeting only health and long‐term care workers yielded very few results; and (2) we felt that broadening to other populations and contexts could unveil important lessons learned and strategies that could be relevant to the health and long‐term care sectors. Below, we have outlined the inclusion criteria used in this review.

### 3.1 Participants

This review will consider only COVID‐19 vaccine educational interventions delivered to adult populations and will exclude any education developed and delivered to children or adolescent populations.

### 3.2 Concept

This review will focus only on interventions that educated participants about any of the COVID‐19 vaccines and the process of receiving any COVID‐19 vaccination. We will include studies and reports that describe both informally and formally delivered educational interventions pertaining to any of the COVID‐19 vaccines. With respect to both informal and formal delivery of education, we will define *education* as an informative action, interaction, or intervention. More specifically, we will characterize *formal education* as education that is guided or systematic (Feng et al., 2017). For example, formal education is described as being introduced through a rigid curriculum and delivered in ‘formal institutions’ (Feng et al., 2017). Whereas, *informal education*, as defined by Spaan and colleagues, is any unstructured or opportunistic interactions that take place outside of formal training settings and are ‘in the control of the learner’ (Spaan et al., 2016). Therefore, we anticipate identify studies that discuss workshops or presentations, as well peer‐to‐peer educative interactions. Studies that describe exclusively educational resources or tools with no clearly described delivery interaction between facilitator and audience or participant will not be included in this review. These include generic emails, information handouts, or online e‐learning requirements, for example. Finally, reports or studies focused on non‐COVID‐19 related vaccines will also be excluded.

### 3.3 Context

As COVID‐19 is a global pandemic, this review will include studies and reports outlining COVID‐19 vaccine educational interventions from anywhere in the world. However, we excluded any national or mass vaccination campaigns, as well as any prompting or messaging interventions, as our focus is to better understand the delivery of the education itself and the potential for any interactions between the facilitator and the participants. In addition, we wanted to ensure that any of the educational characteristics we captured in this review were reproducible at an institutional, hospital or long‐term care level, and would allow opportunities for engaged learning at time of delivery.

### 3.4 Types of sources

Studies and reports included in this review will be identified through a comprehensive search of various electronic databases, grey literature sources and reference scanning of relevant studies. All study designs and papers will be considered, including primary research studies, systematic reviews, case studies, short research reports, editorial comment, rapid communications, letters to the editor, and opinion papers. We chose to include a breadth of sources to ensure our review could capture preliminary evidence, as well as any documented experiential or narrative reports of COVID‐19 vaccine education delivery.

## 4 METHODS

A scoping review methodology was used as it facilitates an exploratory approach to mapping an emerging concept and a broad range of published and unpublished literature. We followed the Joanna Briggs Institute Evidence Synthesis Reporting Guide for Protocols and the Preferred Reporting Items for Systematic Review and Meta‐Analysis Protocols (PRISMA‐P) statement (Tricco et al., 2018) for the reporting of methods undertaken in this review.

### 4.1 Search strategy

In consultation with an experienced medical information scientist, we will develop and test the search strategies through an iterative process in consultation with the review team. The MEDLINE strategy will be peer reviewed by another senior information scientist before execution using the PRESS Checklist (McGowan et al., 2016). With respect to our search strategy, we will use the Ovid platform to search the following databases: Ovid MEDLINE®, including Epub Ahead of Print, In‐Process & Other Non‐Indexed Citations, Embase Classic+Embase, and APA PsycInfo. We will also search CINAHL (Ebsco) and Web of Science. The strategies will utilize a combination of controlled vocabulary (e.g., ‘COVID‐19’, ‘COVID‐19 Vaccines’, ‘Vaccination Refusal’) and keywords (e.g., ‘nCoV’, ‘vaccination’, ‘confidence’). Vocabulary and syntax will be adjusted across the databases. No language or date limits will be applied but animal‐only records will be removed from the results. Results will be downloaded and deduplicated using EndNote version 9.3.3 (Clarivate Analytics) and uploaded to Covidence. We will carry out a grey literature search of selected sites listed in CADTH's *Grey matters: a practical guide to searching health‐related grey literature* (CADTH, 2021). We will also search the following COVID‐19‐specific resources: Cochrane COVID‐19 Study Register, Covid‐END, L‐OVE, LTCcovid.org, UNCOVER, the COVID‐19 resources from ClinicalTrials.gov and the WHO COVID‐19 Database. Additionally, a manual search of the reference lists of included studies will be conducted to identify any literature that was not captured in the original search. A copy of the proposed MEDLINE strategy appears in Supporting Information: Appendix 1. As this is a living review, we are planning to update the search at 6 months (midway mark) and 1 year following the initial search. Any updates to the search will be done in consultation with experts and our information scientist to ensure it is appropriate and has potential to meaningfully contribute to the literature.

### 4.2 Study selection

Studies will be selected according to pre‐defined criteria, as stated above. Three researchers (ACR, MM, AR) will review all results from relevant searchers as per PRISMA Extension for Scoping Reviews (PRISMA‐ScR) protocols (Tricco et al., 2018). Bibliographic screening will also be used to identify any additional relevant publications. Titles and abstracts will be screened independently by three researchers. Full texts of potentially eligible papers for inclusion will be retrieved and screened, and bibliographies of selected studies will be screened for relevant studies missed during the search process. Any screening conflicts that may arise will be discussed between researchers’ ACR, MM, and AR, should they be unable to reach a consensus another member of the research team (AH) will be brought in to make the final decision. Data will then be extracted from the studies selected for inclusion.

### 4.3 Data extraction

Our approach to data extraction and organization was informed by the *Behavioural Science Principles for Supporting COVID‐19 Vaccine Confidence and Uptake Among Ontario Health Care Workers* (Presseau et al., 2021). Using our data extraction chart developed by the research team, ACR, MM and AR will extract the appropriate data from the final papers included in the scoping review. The extracted data will include, authors, country of study, date of publication, study design, program title, program setting, objectives, key components, key content, delivery format, delivery structure, program facilitators, participants, delivery timeline, evaluation metrics, outcomes, and feasibility assessment. We have included a draft version of our data extraction table in Supporting Information: Appendix 2. The data extraction table will be modified and revised as appropriate throughout the data extraction process. Any modifications to the data extraction table will be documented in the full scoping review.

### 4.4 Data analysis and presentation

Following data extraction, the reviewers will discuss the findings of each study, highlighting any commonalities as well as differences between the included studies. Data will be displayed in a tabular format, accompanied by a narrative summary that describes how the data relates to the scoping review objectives and research questions. Emphasis will be placed on any findings that relate directly to the health and long‐term care workforces. In addition, we will highlight any lessons learned from other populations and contexts that could be applied to COVID‐19 vaccine education interventions intended for health and long‐term care staff. Furthermore, we will identify study characteristics and any gaps in the literature that could merit future exploration. As is common practice in scoping reviews, we plan to include stakeholder consultation throughout our review and will share our findings with our long‐term care partners in Ontario. Finally, a PRISMA flow diagram will be used to outline the screening process of the academic literature.

## 5 POTENTIAL IMPACT

Presently, research and evidence around the measurable impact of various education strategies to improve vaccine confidence is limited. We anticipate this scoping review will contribute to this gap by identifying important characteristics and concepts that could merit future exploration and study. More specifically, while the studies and reports included in this review use a variety of methods, both with and without primary data sources, we anticipate being able to map out how research is being conducted is this area, understand the challenges faced and identify any existing knowledge gaps. Furthermore, while COVID‐19 vaccines have been mandated for health and long‐term care workforces in some jurisdictions (Ontario Government, 2021; Paterlini, 2021), the COVID‐19 context continues to evolve and vaccine requirements continue to be modified. Thus, a better understanding of how to support COVID‐19 vaccine confidence and uptake among these workforces continues to be vital. Finally, these findings could also contribute to education developed for any future booster vaccinations, new COVID‐19 vaccines, or non‐COVID‐19 vaccines.

## CONTRIBUTIONS OF AUTHORS

Content Expertise: AH is an experienced investigator, with content expertise in long‐term care and health care delivery. ACR has clinical expertise in both hospital and long‐term care, with additional experience supporting education in both settings.

Methodological Expertise: VW is an expert on analytic methods in systematic reviews and guidelines focusing on global health and health equity. BS is an experienced information specialist with significant experience working with research teams supporting systematic reviews, rapid reviews, scoping reviews, network meta‐analyses, clinical practice guidelines and health technology assessments.

Statistical Expertise: None. No statistical analysis will be conducted.

Content: ACR, MM, AR, MS, VW, ATH

Systematic review methods: BS, VW

Statistical analysis: N/A

Information Retrieval: ACR, MM, AR, MS

## DECLARATION OF INTEREST

Vivian Welch is editor in chief and interim CEO of the Campbell Collaboration. She was not involved in the editorial process or decision to publish for this manuscript. No other conflicts of interest to declare.

## SOURCES OF SUPPORT

External Sources: This work has been funded in part by the Canadian Institutes of Health Research (Funding reference no. GA3‐177726) and by the Public Health Agency of Canada (PHAC) through the COVID‐19 Immunity Task Force (CITF). Ce projet a été financé en partie par l'Agence de la santé publique du Canada (ASPC) par l'intermédiaire du Groupe de travail sur l'immunité face à la COVID‐19 (GTCI). The views expressed herein do not necessarily represent the views of the Public Health Agency of Canada.

Internal Sources: None

## Supporting information

Supporting information.

Supporting information.
