# Supplementary material for: PROTOCOL: Exploring education to support vaccine confidence amongst healthcare and long‐term care staff amidst the COVID‐19 pandemic: A protocol for a living scoping review
Source: Campbell Syst Rev. 2022 Dec 7;18(4):e1293. doi: 10.1002/cl2.1293 (PMC9728484; doi:10.1002/cl2.1293)
Supplement: Supplementary file 1 — Supporting information. [file CL2-18-0-s001.docx]

Vaccine Confidence

2021 Jul 21

Database: Ovid MEDLINE: Epub Ahead of Print, In-Process & Other Non-Indexed Citations, Ovid MEDLINE® Daily and Ovid MEDLINE® <1946-Present>

Search Strategy:

--------------------------------------------------------------------------------

1 COVID-19/ (93080)

2 SARS-CoV-2/ (72264)

3 Coronavirus/ (4746)

4 Betacoronavirus/ (33209)

5 Coronavirus Infections/ (44978)

6 (COVID-19 or COVID19).tw,kf. (139126)

7 ((coronavirus* or corona virus*) and (hubei or wuhan or beijing or shanghai)).tw,kf. (5051)

8 (wuhan adj5 virus*).tw,kf. (238)

9 (2019-nCoV or 19nCoV or 2019nCoV).tw,kf. (1760)

10 (nCoV or n-CoV or "CoV 2" or CoV2).tw,kf. (53484)

11 (SARS-CoV-2 or SARS-CoV2 or SARSCoV-2 or SARSCoV2 or SARS2 or SARS-2 or severe acute respiratory syndrome coronavirus 2).tw,kf. (54296)

12 (2019-novel CoV or Sars-coronavirus2 or Sars-coronavirus-2 or SARS-like coronavirus* or ((novel or new or nouveau) adj2 (CoV or nCoV or covid or coronavirus* or corona virus or Pandemi*2)) or (coronavirus* and pneumonia)).tw,kf. (18877)

13 (novel coronavirus* or novel corona virus* or novel CoV).tw,kf. (9706)

14 ((coronavirus* or corona virus*) adj2 "2019").tw,kf. (32280)

15 ((coronavirus* or corona virus*) adj2 "19").tw,kf. (5222)

16 (coronavirus 2 or corona virus 2).tw,kf. (17082)

17 (OC43 or NL63 or 229E or HKU1 or HCoV* or Sars-coronavirus*).tw,kf. (3713)

18 COVID-19.rx,px,ox. or severe acute respiratory syndrome coronavirus 2.os. (4589)

19 (coronavirus* or corona virus*).ti. (22355)

20 ("B.1.1.7" or "B.1.351" or "B.1.617" or "B.1.617.2" or "B.1.427" or "B.1.429").tw,kf,rx,px,ox. (527)

21 ("P.1" and (Brazil* or variant?)).tw,kf,rx,px,ox. (1510)

22 ((alpha or beta or delta or gamma or lambda) adj3 variant?).tw,kf. (5923)

23 or/1-22 [COVID-19] (176891)

24 exp Vaccination/ (91203)

25 COVID-19 Vaccines/ (3702)

26 (immunis* or immuniz* or inoculat* or vaccin* or unvaccin*).tw,kf. (538375)

27 or/24-26 [VACCINATION] (548857)

28 23 and 27 [COVID-19 - VACCINATION] (17289)

29 Vaccination Refusal/ (569)

30 ((immunis* or immuniz* or inoculat* or vaccin*) adj5 (accept* or ambivalen* or apprehensive* or concern or concerns or confiden* or nonconfiden* or non-confiden* or disinclin* or distrust* or dubious* or hesitan* or hesitat* or indecisiv* or indispos* or mistrust* or oppos* or readiness* or refus* or reject* or reluctan* or resist* or skeptic* or suspici* or tentativ* or trust* or undecided or uncertain* or unsure* or unwilling* or vacillat*)).tw,kf. (16098)

31 (antivaccine? or anti-vaccine? or antivaccinat* or anti-vaccinat*).tw,kf. (673)

32 *Patient Acceptance of Healthcare/ (30538)

33 or/29-32 [VACCINE HESITANCY ETC] (45958)

34 28 and 33 [COVID-19 - VACCINE HESITANCY ETC] (1370)

35 exp Animals/ not Humans/ (4863263)

36 34 not 35 [ANIMAL-ONLY REMOVED] (1350)

***************************
